# Information on blinding in registered records of clinical trials

**DOI:** 10.1186/1745-6215-13-210

**Published:** 2012-11-15

**Authors:** Roderik F Viergever, Davina Ghersi

**Affiliations:** 1 Department of Health Services Research and Policy, London School of Hygiene and Tropical Medicine, 15-17 Tavistock Place, London WC1H 9SH, UK; 2 Department of Primary and Community Care, Radboud University Nijmegen Medical Center, PO Box 9101, 6500HB, Nijmegen, The Netherlands; 3 NHMRC Clinical Trials Centre, Sydney Medical School, University of Sydney, Sydney, Australia

**Keywords:** Clinical trials, Clinical trials registration, Blinding, Masking, Research policy

## Abstract

Information on blinding is part of the data that should be provided upon registration of a trial at a clinical trials registry. Reporting of blinding is often absent or of low quality in published articles of clinical trials. This study researched the presence and quality of information on blinding in registered records of clinical trials and highlights the important role of data-recording formats at clinical trial registries in ensuring high-quality registration.

## Background

The International Clinical Trials Registry Platform (ICTRP) at the World Health Organization (WHO) provides a single point of access to information on more than 200.000 clinical trials made available by registries around the world [1]. To set a standard for the quality of entries in registered records, the WHO Trial Registration Data Set was established, defining the minimum amount of trial information that must appear in a record [2]. Part of the information that is required on study design consists of information on whether blinding was used and, if so, who was blinded.

We recently reported on the quality of information in a random sample of registered records of clinical trials taken from the ICTRP [3]. In this report, we outline the inconsistencies that we encountered in the use of blinding terminology and highlight the important role of data-recording formats in attaining high-quality trial registration.

## Findings

Our previous study analyzed 731 registered records of clinical trials that were registered between 17 June 2008 and 17 June 2009 at one of nine clinical trial registries around the world [3]. This sample was acquired by taking a random 5% sample from the ICTRP database. We report here on the same sample, with the exception that single-arm trials were excluded because they lacked relevance to blinding. The presence and quality of information on blinding was assessed for 571 records.

For each registered record we denoted: 1) whether there was information on blinding in the registered record; 2). whether the record reported a blinding label and if so, what the blinding label was; and 3) whether the record mentioned who was blinded in the trial, and if so, which groups of individuals were blinded.

Of the 571 records in our study sample, 43 (8%) did not contain any information on blinding, and 212 records (37%) were of trials where there was no blinding (open-label). Of the 316 records (55%) that reported that participants were blinded as part of the trial, 48 records (15%) reported only blinding labels (single-blind, double-blind), 8 (3%) contained information only on who was blinded, and 260 (82%) reported both.

For the 260 records for which both blinding labels and information on who was blinded were present, blinding labels were cross-tabulated with who was blinded (Table 1).

**Table 1:** **Trialists**’ **interpretations of the terms ‘single-blind’ and ‘double-blind’**^**a**^

**Who was mentioned as blinded**	**Trial was labelled as**	
	**Single-blind**	**Double-blind**
Patient, caregiver, data analyst / investigator, outcomes assessor	0	79
Patient, caregiver, data analyst / investigator	0	19
Patient, caregiver, outcomes assessor	0	2
Patient, data analyst / investigator, outcomes assessor	0	9
Caregiver, data analyst / investigator, outcomes assessor	0	1
Data analyst / investigator, outcomes assessor	0	1
Patient, outcomes assessor	0	12
Patient, data analyst / investigator	1	68
Patient, caregiver	0	3
Outcomes assessor	22	0
Data analyst / investigator	16	0
Caregiver	1	0
Patient	26	0
Total	66	194

^a^Records that contained both blinding labels and information on who was blinded mainly originated from two registries. One registry asked for information on the blinding status of subjects, clinicians or therapists, outcome assessors and/or data analysts. The other registry asked for information on the blinding status of subjects, caregivers, outcome assessors and/or investigators. Because of the similarity between these two classifications, for this table, trials that were labeled to the fourth category of ‘data analysts’ on one registry and ‘investigators’ on the other were categorized as the same group.

**Figure 1:**
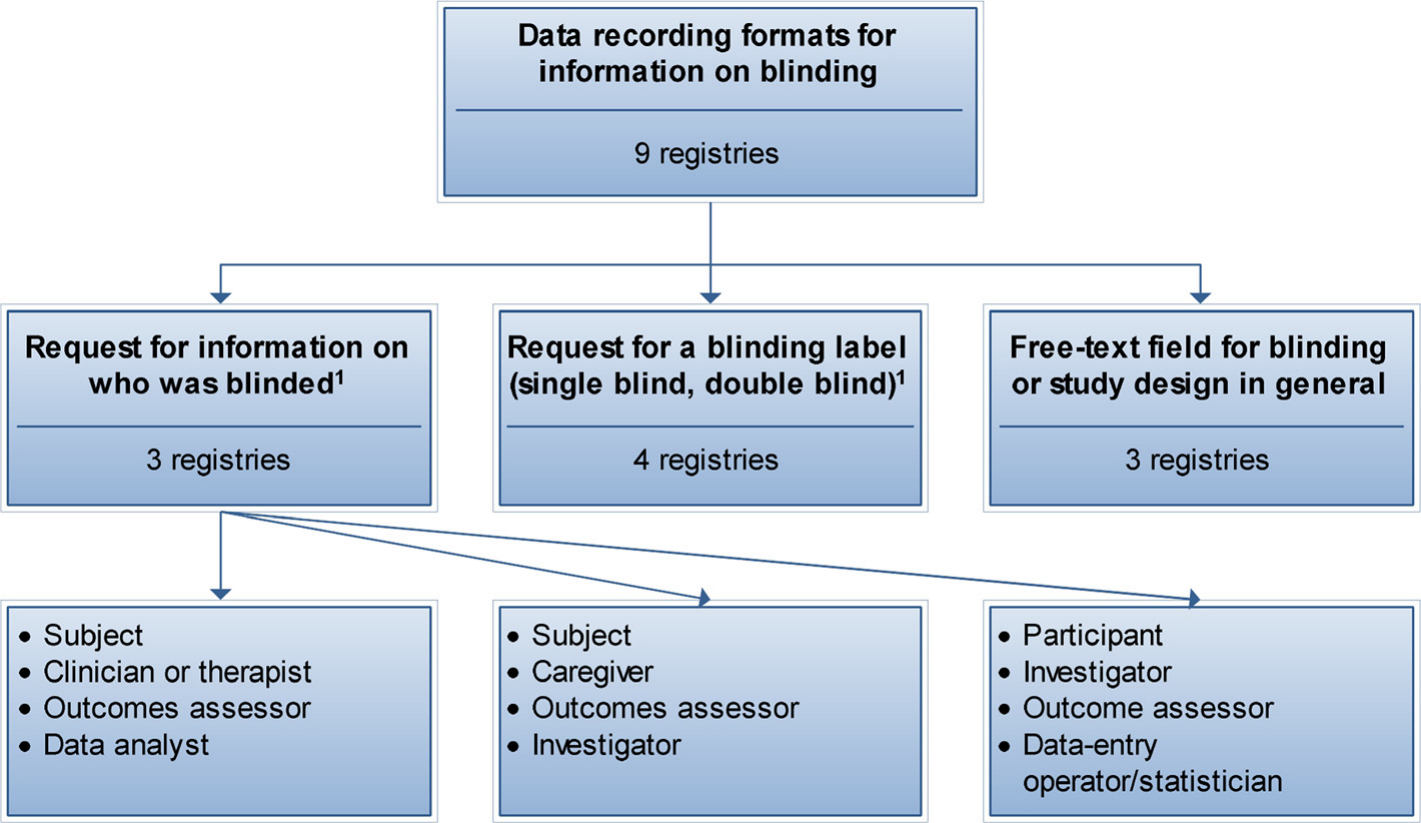
**Data recording formats for information on blinding at the nine registries. **Three registries requested information on who was blinded by asking for the blinding status of specific groups of individuals. The groups of individuals about which information was requested differed per registry. ^1^One registry requested both information on groups and a blinding label.

Data-recording formats for blinding varied across the registries (Figure 1). Records from one of the three registries with free text fields for blinding or study design were less likely to contain any information on blinding, compared with registries that requested information on who was blinded or that requested a blinding label (OR = 23, 95% CI 11 to 48, χ^*2*^ = 123, *P*<0.001). Records from one of the three registries that specifically asked for information on who was blinded were more likely to contain this information, compared with records from registries that only asked for a blinding label or had a free-text field for blinding or study design (OR = 719, 95% CI 91 to 5664, χ^*2*^ = 205, *P*<0.001).

## Discussion

Information on blinding is often not provided in published articles of clinical trials [4], and many trials remain unpublished [5]. Clinical trial protocols offer the most complete resource of information on the study design of trials [6]. Given the current absence of open access to clinical trial protocols, the only other source of information on study design that is publicly available is the registered record of the trial. It is therefore important that information on blinding can be found in the study-design descriptions of registered records of trials. This study shows that this is not always the case. In addition, the sole use of the terms ‘single blinding’ and ‘double blinding’ was found to be common, despite the lack of clarity on their exact meaning. It is a confirmation that these labels should not be used alone, but should be accompanied by information on who was blinded [7-9].

Until recently, the groups of individuals that can potentially introduce a bias into a trial through knowledge of treatment assignments were not clearly defined. The groups on which the registries in our study requested information were not consistent (Figure 1). The 2010 revision to the Consolidated Standards of Reporting Trials (CONSORT) statement has created considerable clarity on this issue by defining five possible groups of people that can be blinded in a trial: participants, healthcare providers, data collectors, outcome adjudicators, and data analysts (See Additional file 1; taken from the CONSORT 2010 statement) [10]. The widespread use of these definitions by clinical trial registries would improve the quality and interpretability of information on blinding across clinical trial records from different registries.

More generally, our findings confirm the pivotal role for data recording formats at clinical trial registries in attaining high quality information in registered records of clinical trials [3,11]. The ICTRP has recognized this and has recently initiated the establishment of International Standards for Clinical Trial Registries. The aim of these standards is to improve the quality of registered data by establishing a minimum requirement for quality control processes performed and data recording practices used by individual clinical trial registries. It is important that the quality of registered trial information continues to be monitored, especially after the introduction of these standards.

## Competing interests

DG was the Team Leader of the International Clinical Trial Registry Platform (ICTRP) of the World Health Organization when this study was conducted. RFV has no competing interests to report.

## Authors’ contributions

Conceived and designed the experiments: RFV DG. Performed the experiments: RFV. Analyzed the data: RFV DG. Wrote the paper: RFV DG. All authors read and approved the final manuscript.

## Funding sources

There are no funding sources to report.

## Supplementary Material

Additional file 1 **Item 11a of the CONSORT 2010 statement reads: ‘Box 4, on blinding terminology, defines the groups of individuals (that is, participants, healthcare providers, data collectors, outcome adjudicators, and data analysts) who can potentially introduce bias into a trial through knowledge of the treatment assignments.’**[10]. Click here for file

## References

[B1] WHO International Clinical Trials Registry Platform (ICTRP) http://www.who.int/ictrp

[B2] WHO Trial Registration Data Sethttp://www.who.int/ictrp/network/trds/en/

[B3] Viergever RF Ghersi D The Quality of Registration of Clinical Trials PLoS One 2011 6 e14701 10.1371/journal.pone.0014701 21383991 PMC3044717

[B4] Hróbjartsson A Pildal J Chan AW Haahr MT Altman DG Gøtzsche PC Reporting on blinding in trial protocols and corresponding publications was often inadequate but rarely contradictory J Clin Epidemiol 2009 62 967 973 10.1016/j.jclinepi.2009.04.003 19635403

[B5] Dwan K Altman DG Arnaiz JA Bloom J Chan AW Cronin E Decullier E Easterbrook PJ Von Elm E Gamble C Ghersi D Ioannidis JP Simes J Williamson PR Systematic review of the empirical evidence of study publication bias and outcome reporting bias PLoS One 2008 3 e3081 10.1371/journal.pone.0003081 18769481 PMC2518111

[B6] Mhaskar R Djulbegovic B Magazin A Soares HP Kumar A Published methodological quality of randomized controlled trials does not reflect the actual quality assessed in protocols J Clin Epidemiol 2012 65 6 602 609 10.1016/j.jclinepi.2011.10.016 22424985 PMC3637913

[B7] Montori VM Bhandari M Devereaux PJ Manns BJ Ghali WA Guyatt GH In the dark: the reporting of blinding status in randomized controlled trials J Clin Epidemiol 2002 55 787 790 10.1016/S0895-4356(02)00446-8 12384193

[B8] Haahr MT Hróbjartsson A Who is blinded in randomized clinical trials? A study of 200 trials and a survey of authors Clinical trials (London, England) 2006 3 360 365 10.1177/1740774506069153 17060210

[B9] Devereaux PJ Manns BJ Ghali WA Quan H Lacchetti C Montori VM Bhandari M Guyatt GH Physician interpretations and textbook definitions of blinding terminology in randomized controlled trials JAMA 2001 285 2000 2003 10.1001/jama.285.15.2000 11308438

[B10] Moher D Hopewell S Schulz KF Montori V Gøtzsche PC Devereaux PJ Elbourne D Egger M Altman DG CONSORT 2010 Explanation and Elaboration: updated guidelines for reporting parallel group randomised trials BMJ 2010 340 c869 10.1136/bmj.c869 20332511 PMC2844943

[B11] Reveiz L Chan AW Krleza-Jerić K Granados CE Pinart M Etxeandia I Rada D Martinez M Bonfill X Cardona AF Reporting of methodologic information on trial registries for quality assessment: a study of trial records retrieved from the WHO search portal PLoS One 2010 5 e12484 10.1371/journal.pone.0012484 20824212 PMC2930852

