# Supplementary material for: Information on blinding in registered records of clinical trials
Source: Trials. 2012 Nov 15;13:210. doi: 10.1186/1745-6215-13-210 (PMC3522538; doi:10.1186/1745-6215-13-210)
Supplement: Additional file 1 — Item 11a of the CONSORT 2010 statement reads: ‘Box 4, on blinding terminology, defines the groups of individuals (that is, participants, healthcare providers, data collectors, outcome adjudicators, and data analysts) who can potentially introduce bias into a trial through knowledge of the treatment assignments.’[10]. [file 1745-6215-13-210-S1.docx]

# Additional file 1

Item 11a of the CONSORT 2010 statement reads: ‘Box 4, on blinding terminology, defines the groups of individuals (that is, participants, healthcare providers, data collectors, outcome adjudicators, and data analysts) who can potentially introduce bias into a trial through knowledge of the treatment assignments.’

References:

Moher D, Hopewell S, Schulz KF, Montori V, Gøtzsche PC, Devereaux PJ, Elbourne D, Egger M, Altman DG: **CONSORT 2010 Explanation and Elaboration: updated guidelines for reporting parallel group randomised trials.** *BMJ* 2010, **340**:c869.

*The CONSORT Statement Website: Item 11a - Blinding*. <http://www.consort-statement.org/consort-statement/3-12---methods/item11a_blinding/> (accessed 14 November 2012).

Box 4 from the 2010 Consort statement:

# Box4: Blinding terminology

In order for a technical term to have utility it must have consistency in its use and interpretation. Authors of trials commonly use the term “double blind” and, less commonly, the terms “single blind”or “triple blind.” A problem with this lexicon is that there is great variability in clinician interpretations and epidemiological textbook definitions of these terms.^169^ Moreover, a study of 200 RCTs reported as double blind found 18 different combinations of groups actually blinded when the authors of these trials were surveyed, and about one in every five of these trials—reported as double blind—did not blind participants, healthcare providers, or data collectors.^170^

This research shows that terms are ambiguous and, as such, authors and editors should abandon their use. Authors should instead explicitly report the blinding status of the people involved for whom blinding may influence the validity of a trial.

Healthcare providers include all personnel (for example, physicians, chiropractors, physiotherapists, nurses) who care for the participants during the trial. Data collectors are the individuals who collect data on the trial outcomes. Outcome adjudicators are the individuals who determine whether a participant did experience the outcomes of interest.

Some researchers have also advocated blinding and reporting the blinding status of the data monitoring committee and the manuscript writers.^160^ Blinding of these groups is uncommon, and the value of blinding them is debated.^171^

Sometimes one group of individuals (such as the healthcare providers) are the same individuals fulfilling another role in a trial (such as data collectors). Even if this is the case, the authors should explicitly state the blinding status of these groups to allow readers to judge the validity of the trial.

References:

160 Gøtzsche PC. Blinding during data analysis and writing of manuscripts. Control Clin Trials. 1996;17:285–90.

169 Devereaux PJ, Manns BJ, Ghali WA, Quan H, Lacchetti C, Guyatt GH. In the dark: physician interpretations and expert definitions of blinding in randomized controlled trials. JAMA. 2001;285:2000–3.

170 Haahr MT, Hróbjartsson A. Who is blinded in randomized clinical trials? A study of 200 trials and a survey of authors. Clin Trials 2006;3:360–5.

171 Meinert CL. Masked monitoring in clinical trials—blind stupidity? N Engl J Med 1998;338:1381–2.
